# Paternal Age Amplifies Cryopreservation-Induced Stress in Human Spermatozoa

**DOI:** 10.3390/cells13070625

**Published:** 2024-04-04

**Authors:** Silvia Pérez Casasús, Francesca Paola Luongo, Alesandro Haxhiu, Martina Orini, Giorgia Scupoli, Laura Governini, Paola Piomboni, Jose Buratini, Mariabeatrice Dal Canto, Alice Luddi

**Affiliations:** 1 Department of Molecular and Developmental Medicine, University of Siena, 53100 Siena, Italy; s.perezcasasus@student.unisi.it (S.P.C.); luongofrancescapaola@gmail.com (F.P.L.); alesandro.haxhiu@student.unisi.it (A.H.); orini.martina@gmail.com (M.O.); giorgia.scupoli@gmail.com (G.S.); laura.governini@unisi.it (L.G.); luddi@unisi.it (A.L.); 2 Biogenesi Reproductive Medicine Center, Istituti Clinici Zucchi, 20900 Monza, Italy; jburatini@eugin.it (J.B.); dalcanto@biogenesi.it (M.D.C.)

**Keywords:** APA, sperm cryopreservation, mitochondrial functionality, acrosomal reaction, mtDNAcn, tyrosine phosphorylation

## Abstract

The global fall in male fertility is a complicated process driven by a variety of factors, including environmental exposure, lifestyle, obesity, stress, and aging. The availability of assisted reproductive technology (ART) has allowed older couples to conceive, increasing the average paternal age at first childbirth. Advanced paternal age (APA), most often considered male age ≥40, has been described to impact several aspects of male reproductive physiology. In this prospective cohort study including 200 normozoospermic patients, 105 of whom were ≤35 years (non-APA), and 95 of whom were ≥42 years (APA), we assessed the impact of paternal age on different endpoints representative of sperm quality and cryopreservation tolerance. Non-APA patients had superior fresh semen quality; DNA fragmentation was notably increased in APA as compared to non-APA individuals (21.7% vs. 15.4%). Cryopreservation further increased the DNA fragmentation index in APA (26.7%) but not in non-APA patients. Additionally, APA was associated with increased mtDNAcn in both fresh and frozen/thawed sperm, which is indicative of poorer mitochondrial quality. Cryopreservation negatively impacted acrosome integrity in both age groups, as indicated by reduced incidences of unreacted acrosome in relation to fresh counterparts in non-APA (from 71.5% to 57.7%) and APA patients (from 75% to 63%). Finally, cryopreservation significantly reduced the phosphorylation status of proteins containing tyrosine residues in sperm from young males. Therefore, the present findings shed light on the effects of paternal age and cryopreservation on sperm quality and serve as valuable new parameters to improve our understanding of the mechanisms underlying sperm developmental competence that are under threat in current ART practice.

## 1. Introduction

A substantial reduction in semen quality has been documented over the past few decades [1,2,3,4,5]. The worldwide decline in male fertility is a complex phenomenon influenced by various factors, such as environmental endocrine disruptors, lifestyle, obesity, stress, and increasing paternal age [6,7]. This latter is attributable to factors such as increased life expectancy, societal expectations, and delayed marriage. The accessibility to assisted reproductive technologies (ART) has enabled older couples to conceive, contributing to the rise in average paternal age at first childbirth. In contrast to strong efforts towards the understanding of the impact of maternal age on oocyte quality, the impact of paternal age on sperm developmental competence has been neglected and remains unclear.

Advanced paternal age (APA), referring to men conceiving at the age of 40 or beyond [8], impacts various aspects of male reproductive physiology. Indeed, APA has been associated with altered testicular function [9], endocrine alterations [10], modifications in sperm parameters [11,12], compromised sperm DNA integrity, changes in telomere length [13], increased de novo mutation rate [14], modifications in chromosomal structure [15,16], and epigenetic changes [17]. A significant body of evidence consistently indicates a decrease in semen volume, motility, and the proportion of morphologically normal sperm with advancing age. Overall, the most consistent findings include APA’s negative correlation with semen volume (7 of 9 studies) and motility (9 of 10 studies) as well as positive correlation with DNA fragmentation (11 of 11 studies) [4,18,19,20]. Among others, a study involving 277 normozoospermic men revealed a significantly higher DNA Fragmentation Index (DFI) percentage in men older than 40 years compared to younger men [21]. However, conflicting findings were reported by Winkle et al. [22], who found no significant associations between male age, DNA fragmentation, and semen parameters. The mechanisms behind age-related DNA fragmentation are not entirely clear, but oxidative stress and inefficient apoptosis are believed to be significant contributing factors. Poor semen parameters have also been linked to high mitochondrial DNA copy number (mtDNAcn) [23,24].

Preserving sperm via cryopreservation is an effective method to preserve male fertility, routinely applied in cases of chemotherapy, radiotherapy, testicular surgery, and assisted reproduction [21,22]. Moreover, the adverse effects of sperm cryopreservation on the sperm DNA fragmentation index (DFI) have been extensively documented in the literature [2,15,25,26]. However, despite the effectiveness of cryopreservation, freezing and thawing steps have negative effects on the sperm membrane, acrosome, and DNA integrity, as well as sperm motility and vitality [27,28]. Cryopreservation induces both cryo- and osmotic stress, leading to an increase in the production of reactive oxygen species (ROS) and disruption of the balance in antioxidant defense systems [26,27]. This is why only about 50% of spermatozoa survive the freezing and thawing procedures [25]. Apart from the injuries caused by cryopreservation itself, it triggers premature changes that resemble capacitation [29]. These molecular modifications, known as cryo-capacitation, imitate the typical capacitation process in sperm, leading to a reduced lifespan and diminished fertilization potential. Elevated concentrations of intracellular Ca^2+^ ions have been associated with cryo-capacitation. The interplay between age-related sperm quality decline and cryopreservation-induced stress is mostly unknown and deserves robust investigation in order to provide new reliable/valuable parameters for the improvement in medical strategies aiming at preserving or optimizing male fertility. Sperm parameters, fertility, and male sexual function can all be significantly impacted by age. There are undoubtedly a lot of unanswered questions concerning male aging and fertility, even in light of the facts covered in this article. A deeper comprehension of the modifications to the male reproductive axis and its effects on all aspects of male fertility in relation to age will be possible with additional research.

Considering these observations, the purpose of this study was to explore whether APA exacerbates the stress induced by cryopreservation in sperm. Herein, we tested the hypothesis that APA is associated with a greater impact of cryopreservation on key biological processes essential for sperm’s developmental competence, such as acrosomal reaction (AR), tyrosine phosphorylation levels, DNA integrity, and mitochondrial functionality.

## 2. Materials and Methods

**Figure 1:**
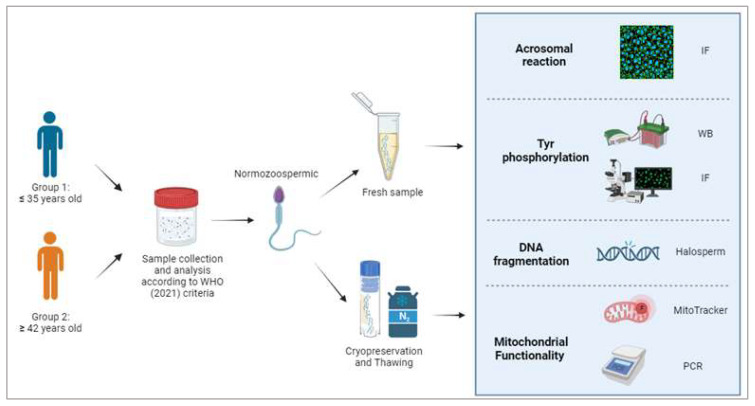
Design of the study. Sample collection and analysis was performed according WHO manual 2021 [30] IF: immunofluorescence analysis; WB: Western blot analysis.

The flowchart of this study is summarized in Figure 1.

### 2.1. Semen Collection and Treatment

This prospective multicentric study was based on a cohort of 200 consecutive men undergoing fertility investigation from January to October 2023 at the Unit of Medically Assisted Reproduction, Siena University Hospital and the Biogenesi Reproductive Medicine Center, Istituti Clinici Zucchi, Monza, Italy. All participants provided written consent for their involvement in the study, and approval for the research was granted by the local Institutional Review Board (approval ID: CEAVSE protocol number 18370, 2 October 2020). All patients underwent a detailed medical history and physical examination, and those exhibiting potential underlying factors associated with male infertility, such as varicocele, cryptorchidism, hormonal imbalances, congenital genital tract anomalies, or prior treatments with potential impacts on fertility, such as cancer therapy, were excluded from the study. Sperm samples were collected via masturbation after a period of abstinence lasting from 2 to 5 days. Semen analysis was conducted within 30 min after fluidification, according to WHO 2021 guidelines, with two separate blinded observers performing the assessment (the reported data represents the average of the two observations). An amount of 10 µL of each semen sample was used for analysis according to the WHO guidelines [30]. An unbiased observer assessed seminal parameters, and the results were independently verified by another observer to ensure quality control. Briefly, 10 µL of semen were loaded in a Makler chamber, and sperm concentration and motility were evaluated by a blinded observer; total sperm, motile, and non-motile sperm were counted in 10 squares. Vitality was measured by mixing semen and eosin 1:1 (*v*:*v*); after 30 s of incubation, sperm were smeared on a slide and examined in a light microscope (400×). Sperm concentration, motility, morphology, and vitality were assessed for each sample, and only those individuals with normal sperm parameters (n = 200) were included in the study. Normozoospermic patients were included in the following analyses once assigned to 2 groups based on their age: the non-APA group comprising patients ≤35 y (n = 105) and the APA group comprising patients ≤42 y (n = 95).

### 2.2. Sperm Cryopreservation and Thawing

Semen samples from each donor were split into two aliquots: one was utilized as a fresh semen sample, while the other underwent a cryopreservation/thawing procedure.

For sperm cryopreservation, an equal volume of cryoprotector solution (Freezing Medium, Fujifilm, Rome, Italy) was added drop by drop to the sperm pellet (volume/volume). Cryotubes containing sperm were firstly placed at 4 °C for 60 min and then transferred to −20 °C for an additional 20 min before being immersed in liquid nitrogen. Sperm samples were thawed by placing them immediately at 37 °C. After complete thawing, the cryoprotectant was removed by adding PBS 1× before centrifugation for 10 min at 500× *g*.

Sperm analysis, following WHO 2021 guidelines, was then repeated using 10 μL of each selected sample, while the remaining sample was stored for subsequent experiments.

### 2.3. Acrosomal Staining

For each semen condition (fresh or freeze/thawed), the assessment of the acrosomal status was conducted using Pisum sativum agglutinin (FITC-PSA), labeled with fluorescein isothiocyanate, according to a previously published method [31]. Fluorescence images were captured using the Leica AF6500 Integrated System for Imaging and Analysis, manufactured by Leica Microsystems in Wetzlar, Germany and operated with the LAS AF 4.0 software. Subsequently, the signal intensity was quantified using Image-J software 1.54i (U.S. National Institutes of Health in Bethesda, MD, USA). A minimum of 200 sperm per patient was counted to determine the number of sperm that were acrosome-reacted or intact.

### 2.4. Western Blotting

For Western blot analysis, sperm samples were washed with ice-cold PBS and subsequently lysed as described in a precedent study with some modification [32] using lysis buffer (8 M urea, 4% CHAPS, 40 mM Tris base, 65 mM dithioerythritol, and trace amounts of bromophenol blue) supplemented with Protease Inhibitor (dilution 1:100) and phenylmethylsulphonyl fluoride (PMSF; dilution 1:200). The protein concentration was determined using a BSA Bradford assay. Then, 30 µg of protein from each sample was separated via electrophoresis, subsequently transferred onto a nitrocellulose membrane, and incubated overnight at 4 °C with the primary antibody (Supplementary Table S1). It was then subjected to three washes before being exposed to the secondary antibody (Supplementary Table S1) for 1 h at RT. Following three additional washes in PBS-Tween 20 0.1%, the immunostained bands were visualized via chemiluminescence using ImageQuant LAS 4000 (GE Healthcare, Chicago, IL, USA).

### 2.5. Immunofluorescence Staining

Immunofluorescence analysis was carried out following a previously published protocol [33]. The list of antibodies used for the staining is available in Supplementary Table S1. The sperm samples were examined using the Leica AF6500 Integrated System for Imaging and Analysis (Leica Microsystems in Wetzlar, Germany) and operated with the LAS AF 4.0 software. Staining patterns were analyzed and classified as reported in the literature [30,34].

### 2.6. Assessment of Sperm DNA Fragmentation

Sperm DNA fragmentation was evaluated using the sperm chromatin dispersion test (SCD test; Halosperm G2^®^ assay, Halotech DNA SL, Madrid, Spain). Following this process, the slides were stained, and at least 300 sperm from each sample were examined to assess the variability in DNA fragmentation levels (DFLs) [31].

### 2.7. Mitochondrial Membrane Potential Assay

MitoTracker staining was employed to evaluate sperm mitochondrial membrane potential (MMP) in the two distinct experimental groups, following a validated procedure [35]. MitoTracker, which selectively labels the midpiece of sperm with active mitochondria, serves as a reliable tool for tracking mitochondrial membrane potential (MMP) in sperm samples [36]. To counterstain the nuclei 4′,6-diamidino-2-phenylindole (DAPI) was utilized. Sperm were classified based on staining intensity and midpiece patterns. A minimum of 200 sperm from at least ten different fields were examined for each sample.

### 2.8. Determination of mtDNA Copy Number

Sperm DNA extraction was conducted using a protocol previously published by Darbandi et al. [37]. In summary, the spermatozoa were incubated with a Lysis Buffer for 15 min at room temperature, followed by centrifugation. The resulting pellet was subjected to treatment with TRIzol and proteinase K. After an overnight incubation at 56 °C, chloroform was added to create three phases (DNA-RNA and proteins), and DNA was collected from the upper phase. Cold ethanol and 3 M sodium citrate were added for DNA sedimentation, followed by washing and rehydration. The final DNA pellet was obtained after centrifugation and washing steps. The purity of RNA was assessed by n NanoDrop^®^ ND-100 (Thermo Fisher Scientific, Waltham, MA, USA).

The relative mtDNA copy numbers were calculated by real-time qPCR and normalized via the quantification of the nuclear DNA. The sequence of primers for nuclear gene microglobin (*Nβ2*) were FW 5′-TGC TGT CTC CAT GTT TGA TGT ATC T-3′ and REV: 5′-TCT CTG CTC CCC ACC TCT AAG T-3′. The primers for the mtDNA MT-ND1 gene were FW 5′-GTC AAC CTC GCT TCC CCA CCC T-3′ and REV 5′-TCC TGC GAA TAG GCT TCC GGC T-3′. All amplification reactions were conducted in triplicate by qRT-PCR on a CFX connect Real-Time PCR Detection System (Bio-Rad Laboratories, Berkeley, CA, USA) using SsoFast EvaGreen Supermix (Bio-Rad Laboratories). The PCR mix consisted of Green Master Mix FAST ROX 2X (Genaxxon bioscience GmbH, Ulm, Germany), primers 1×, DNA (10 ng), and water. The PCR conditions were 3 min at 95 °C, followed by 40 cycles of denaturation at 95 °C for 10 s, annealing at 60 °C for 10 s, and primer extension at 72 °C for 20 s. The Ct values for the Nβ2 microglobin gene and the MT-ND1 gene were ascertained for each individual in a single quantitative PCR run. Ct values serve as a metric for the input copy number, and differences in Ct values are utilized to quantify the mtDNAcn relative to the Nβ2 microglobin gene. This is calculated using the equation for relative copy number (Rc): Rc = −1/2 ∆Ct, where ∆Ct represents the difference between the Ct values of ND1 (Ct_ND1) and Nβ2 microglolin (Ct_Nβ2 microglobin).

### 2.9. Statistical Analysis

Statistical analysis was carried out with GraphPad Prism 9.0 (GraphPad Software, San Diego, CA, USA). Unpaired *t*-test with Welch’s correction for analysis of sperm parameters. One-way ANOVA was used to compare acrosome staining, results from Western blot, and DNA fragmentation in the two groups of samples. A chi-square test of independence was performed to examine the acrosomal integrity in fresh and cryopreserved sperm. Data are reported as mean ± standard deviation (SD). Statistical significance was set at *p* < 0.05.

## 3. Results

### 3.1. Analysis of Sperm Parameters

Table 1 summarizes the main seminal parameters in the two study groups: the non-APA group comprising patients under 35 years old and the APA group comprising patients with more than 42 years. Significant differences were observed in seminal volume (*p* = 0.0219), total number of spermatozoa (*p* = 0.0067), progressive motility (*p* = 0.0231), and percentage of morphologically normal sperm (*p* = 0.0036). However, no statistically significant differences were observed in the number of spermatozoa per mL, motility, and sperm vitality. Additionally, post-cryopreservation motility was not significantly different between the two groups (*p* = 0.1249), with the non-APA group having a mean of 8.556 ± 8.801% and the APA group having a mean of 5.638 ± 6.298%.

### 3.2. DNA Fragmentation

**Figure 2:**
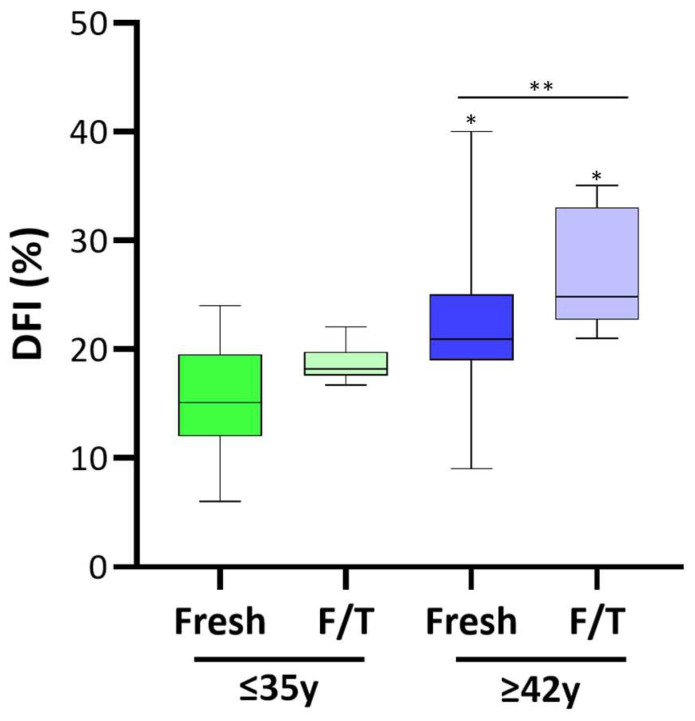
Effect of APA and cryopreservation on the sperm DNA fragmentation Index. Data obtained with the Halosperm kit are plotted as box–whisker plots, where boxes show the interquartile range with median values, and whiskers represent min and max confidence intervals. Number of analyzed samples: ≤35 years fresh and fresh and thawed (F/T) = 23; ≥42 years fresh and fresh and thawed (F/T) = 25. Significance * *p* < 0.05; ** *p* < 0.01.

As illustrated in Figure 2, the mean value of the DNA fragmentation index was similar in fresh and frozen/thawed semen from young men (mean 15.4% vs. 18.4%; *p* > 0.05). The mean percentage of spermatozoa exhibiting DNA fragmentation was significantly elevated in fresh semen from men aged over 42 years compared to those younger than 35 years (15.4% and 21.7%, respectively, *p* < 0.05). Noteworthy, in contrast with the results observed in men younger than 35, DFI significantly increased with cryopreservation in men older than 42 (21.7% vs. 26.7%, *p* < 0.05).

### 3.3. Assessment of Mitochondrial Functionality

**Figure 3:**
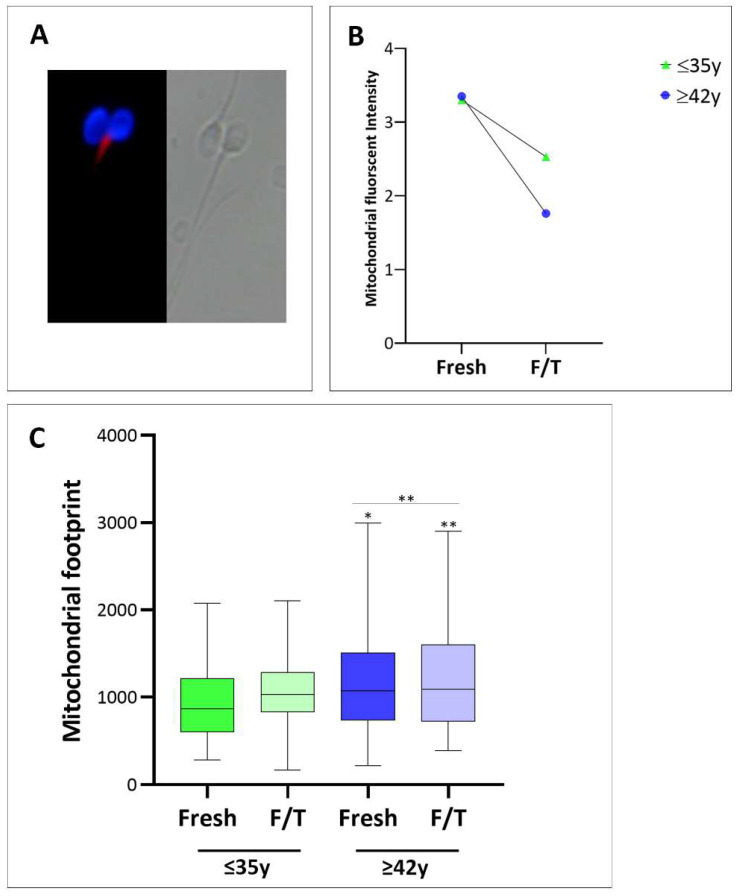
(**A**) Representative bright field and fluorescence images of MitoTracker staining showing a spermatozoon with high intensity of mitochondrial red fluorescence next to a spermatozoon with no signal (**right**). Scale bar 10 µm. (**B**) Graphical representation of mitochondrial staining intensity measured with the Software ImageJ 1.54i. ≤35 years old men, green triangle; ≥42 years old men, blue dot. A total of 22 semen samples for each group were analyzed during the experiment. (**C**) Graphical representation of mitochondrial footprint measured with the Software ImageJ 1.54i. Data are plotted as box–whisker plots, where boxes show the interquartile range with median values, and whiskers represent min and max confidence intervals. Significance * *p* < 0.05, ** *p* < 0.01.

To evaluate MMP in both study groups, MitoTracker-positive sperm were quantified in each sample (Figure 3A). The mean percentage of sperm stained with MitoTracker was comparable in the non-APA and APA groups, both in fresh samples and after cryopreservation. This value ranges between 90% and 98%.

However, when we measured the signal intensity using Mitochondrial Network Analysis (MiNA), we observed that, while the fluorescence intensity in the fresh samples was comparable, there was a statistically significant reduction (*p* < 0.01) in the staining intensity of frozen/thawed sperm compared to the fresh samples. Notably, sperm mitochondria of men older than 42 years showed a 50% reduction in mean fluorescence intensity as compared to individuals younger than 35 (2.5 ± 1.3 and 1.8 ± 0.5, respectively; *p* < 0.001) (Figure 3B).

We conducted a further evaluation of mitochondrial network integrity in all sperm samples (both fresh and thawed). As depicted in Figure 3A, the upper and lower images serve as representative examples of mitochondrial networks in fresh and frozen/thawed sperm. The quantified mitochondrial footprint, presented in Figure 3C, represents the area or volume of the image occupied by the mitochondrial signal. The mitochondrial footprint is significantly larger in thawed sperm from men in the APA group compared to fresh sperm or sperm from younger men (* *p* < 0.05). This observation of an increased mitochondrial footprint lets us hypothesize that mitochondria are likely over-fused due to altered fusion and fission events associated with cryopreservation.

**Figure 4:**
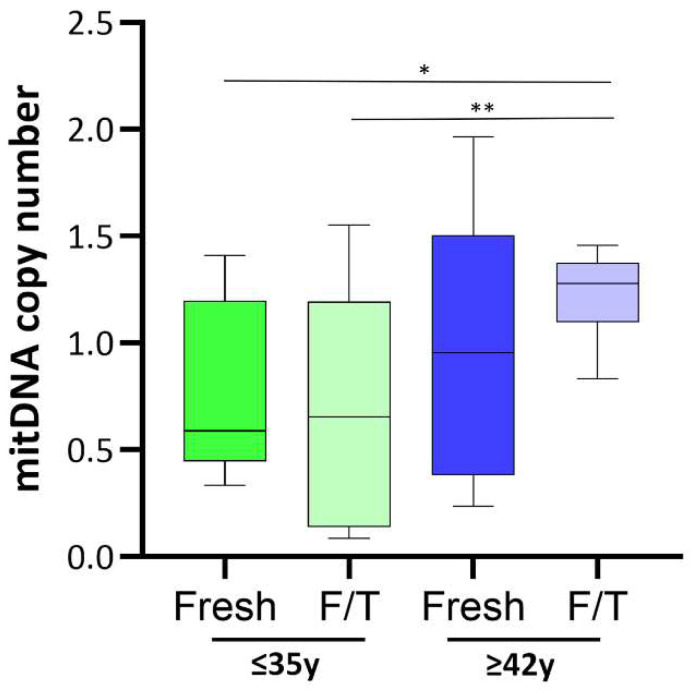
Relative mtDNAcn in the different study groups. This is computed utilizing the formula for relative copy number (Rcn): Rcn = −1/2 ∆Ct, where ∆Ct denotes the disparity between the Ct values of ND1 (Ct_ND1) and Nβ2 microglobin (Ct_Nβ2 microglobin). * *p* < 0.05 ** *p* < 0.001.

Additionally, since the mitochondrial fusion/fission process finely regulates mtDNA synthesis, the mtDNAcn was assessed. As shown in Figure 4, in fresh semen, the median value of mtDNAcn was higher in old men compared to younger, despite this datum does not reach statistical significance. Interestingly, the mtDNAcn in frozen/thawed sperm from the APA group was significantly increased compared to frozen/thawed sperm from the non-APA group (0.57; *p* < 0.01),

### 3.4. Effect of Advanced Male Age on Cryo-Capacitation

**Figure 5:**
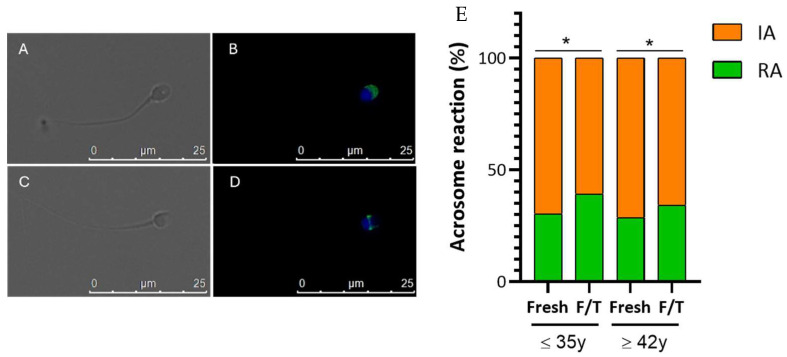
Fluorescence patterns of human spermatozoa stained with PSA (green) for the analysis of acrosome status. DAPI (blue) was used to label sperm in the nucleus. Effect of APA and cryopreservation on acrosome status. (**B**,**D**). Images (**A**,**C**) Differential interference contrast. Scale Bars: 25 µm. (**E**) Percentage of acrosome reaction in non-APA group (fresh and frozen) and APA group (fresh and frozen). A total of 14 semen samples for the non-APA group and 10 samples for the APA group were analyzed during the experiment. IA (orange): intact acrosome cells; RA (green): reacted Acrosome cells. Values are mean percentage ± SD. * *p* < 0.05.

Figure 5 illustrates the characteristic staining pattern of intact acrosome (A,B) and reacted (C,D) acrosome. In each experimental condition, the quantity of sperm exhibiting intact or reacted acrosomes was assessed, revealing a significant decrease (*p* < 0.01) in sperm acrosome integrity after cryopreservation. Specifically, the percentage of intact acrosomes in the non-APA group was 71.5% in fresh samples and 57.7% in frozen/thawed sperm. In the APA group, the corresponding percentages were approximately 75% in fresh samples and 63% in frozen samples (Figure 5E). A chi-square test of independence was performed to examine the relation between male age and the acrosome integrity in fresh and cryopreserved sperm. While the relation between these variables was not significant for fresh sperm, it became significant in frozen/thawed sperm, where the % of intact acrosome significantly decreased in older men X^2^ = 4.3, *p* < 0.001.

**Figure 6:**
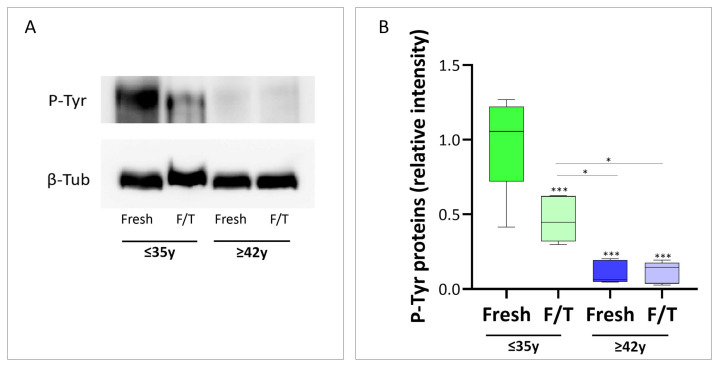
Representative image of WB membrane of tyrosine phosphorylation level in non-APA and APA groups. (**A**) Representative image of Western blot analysis of P-Tyrosyn in both groups. β-tubulin was used as a loading control. A total of 12 samples from different patients were used for this experiment, 6 for each group. (**B**) Whisker-plot graph showing the overall relative intensity of the spots as determined by computer-assisted densitometric analysis. Data show the means ± SD of relative units (RU). Significance * *p* < 0.05, *** *p* < 0.001.

Capacitation-like changes, such as tyrosine phosphorylation, have been demonstrated during cryopreservation [29,38]. Because of this, we compared the phosphorylation status of proteins containing tyrosine residues in the cryopreserved and fresh samples, both in young and old males. Samples from younger men exhibited a band at approximately 200 kDa, corresponding to the family of proteins with tyrosine-phosphorylated residues; this band was fairly detectable in the sperm from old men (Figure 6A). The relative quantification of the spots confirmed the low abundance of P-Tyr proteins in sperm from males of advanced age (*p* < 0.001) but also demonstrated that cryopreservation significantly reduced the phosphorylation status of proteins containing tyrosine residues in sperm from young males (*p* < 0.001) (Figure 6B).

There were no significant changes within the fresh and frozen samples of the APA group.

**Figure 7:**
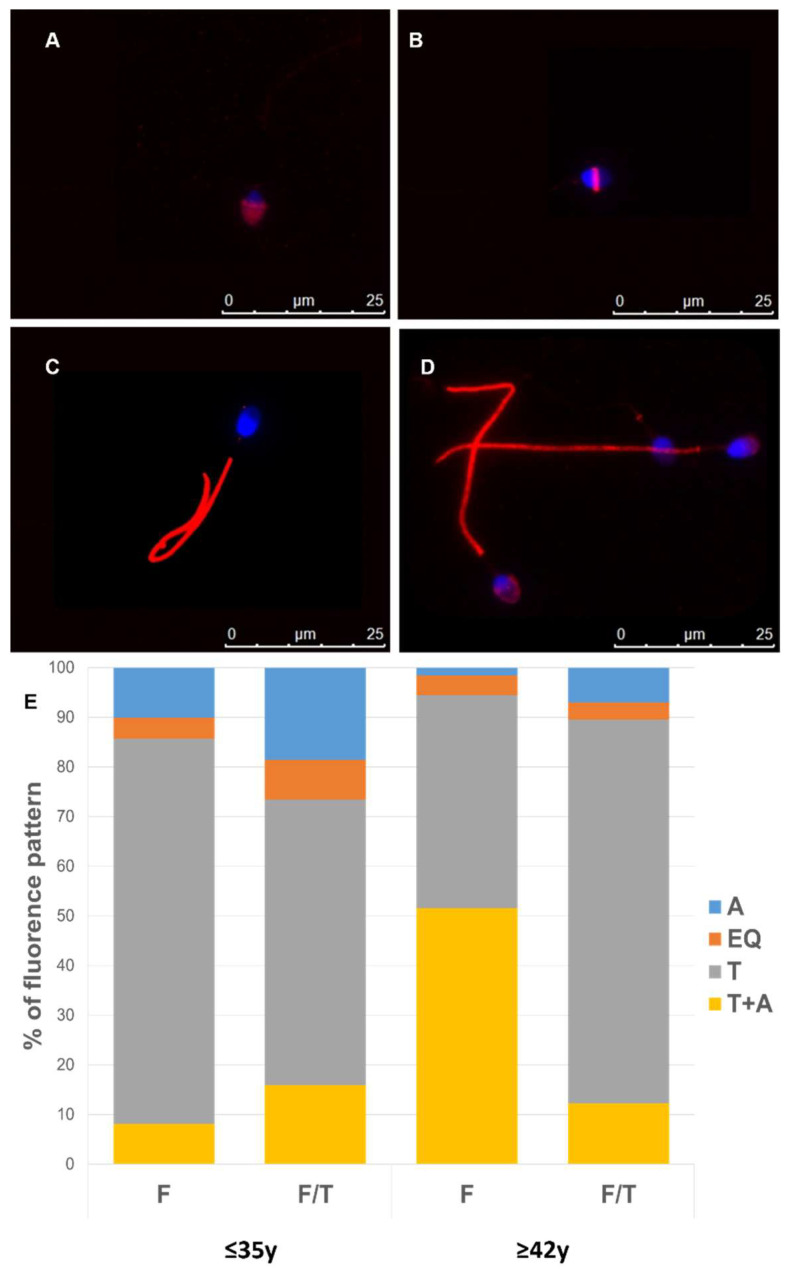
(**A**–**D**) Representative image displaying distinct immunofluorescence localization of phosphotyrosine residues in human sperm using anti-phosphotyrosine antibody (red). Scale bars: 25 µm. (**E**) Sperm phosphotyrosine stainings were classified according to their fluorescence patterns, and the percentage of each pattern was subsequently calculated for each patient. The data represent the mean proportion of each pattern (±SEM). A total of 200 sperm were assessed in each sample. A, acrosome; EQ, equatorial segment; T, tail; T + A, combined staining in the tail and in the acrosome.

To conduct a more comprehensive analysis, we examined the changes in the distribution of phosphotyrosine in both fresh and frozen/thawed human sperm cells, according to male age. The phosphorylated tyrosine residues were identified in the following regions: (1) the acrosomal cap, (2) the equatorial segment, (3) the tail, and (4) the combination of the tail and acrosome (Figure 7). We examined the patterns of phosphotyrosine expression in males from APA and non-APA groups, observing age-related alterations in expression patterns reflected by their varied percentages. Noteworthy, in fresh samples, a reduced number of spermatozoa of old males showed tyrosine phosphorylation in the tail. In frozen/thawed sperm, this percentage significantly decreased in sperm from young males, while it significantly increased in the older ones (Figure 7E).

## 4. Discussion

Sperm cryopreservation is a fundamental procedure for both male fertility preservation and subfertility treatment via ART. A huge amount of data has demonstrated that cryopreservation affects sperm competence. The most frequently reported consequence is a reduction in sperm motility, but it also causes considerable harm to sperm chromatin, morphology, and membrane integrity in both fertile and infertile men [39,40,41]. In the present study, we demonstrate that advanced paternal age amplifies the detrimental effects of cryopreservation, reducing the quality of frozen/thawed spermatozoa.

Several studies have assessed the association between paternal age and sperm DNA fragmentation. Two of them reported a significantly higher sperm DFI in men aged over 40 in comparison to their younger counterparts [42,43]. In contrast, a third study did not find a robust association between male age, DNA fragmentation, and semen parameters [22]. Firstly, in our cohort, we observed a noteworthy rise in sperm DFI with paternal age. The literature extensively documents the negative effects of cryopreservation on sperm DNA fragmentation index [18,26,40,44], although a universal consensus is lacking. Indeed, another study has suggested that there are no significant differences in sperm DFI levels between fresh and cryopreserved semen samples [45]. In this regard, it is appropriate to emphasize the absence of a universally accepted definition for an abnormal DNA fragmentation threshold, along with standardized assays.

We further verified that DNA fragmentation is not significantly increased in frozen/thawed sperm of young men. Nevertheless, our findings indicate that the cryopreservation process leads to a significant increase in sperm DFI in APA sperm. Therefore, we speculate that the vulnerability to cryopreservation DNA damage increases with paternal age. It has been hypothesized that the heightened level of DNA fragmentation observed in older men may stem from increased exposure to DNA damage induced by oxidative stress in their reproductive systems over their lifetime [46].

This study also provides evidence of the impact of APA on mitochondrial activity during sperm cryopreservation. While the MMP in fresh samples is similar between age groups, cryopreserved sperm from men over 42 years exhibit half the mean fluorescence intensity as compared to younger individuals. Recent studies indicate that cryopreservation causes a decline in overall sperm quality and functionality by disrupting mitochondrial ultrastructure and function; additionally, it has been demonstrated that mitochondrial activators may protect against the adverse effects of semen cryopreservation [47,48]. The analysis of mitochondrial network integrity revealed a significantly larger mitochondrial footprint in thawed sperm from older men, suggesting a potential over-fusion of mitochondria, possibly due to altered fusion and fission events associated with cryopreservation. It has been reported that to prevent the formation of mitochondria lacking DNA, mitochondrial fusion facilitates the synthesis of mtDNA via processes involving ROS-triggered, recombination-mediated replication [49].

Consistent with this finding, we observed an increase in mtDNAcn in APA as compared to non-APA sperm (despite not being significant in our study cohort) and a significant impact on mtDNAcn specifically associated with cryopreservation in older men. Previous studies demonstrated a strong inverse association between sperm mtDNAcn and semen quality [23], as well as fertilization competence [50], indicating that mtDNAcn could be a useful biomarker of sperm quality [23,24]. Based on our results, we speculate that cryopreservation may induce mitochondrial fusion, which elicits mtDNA synthesis by facilitating ROS-triggering and recombination-mediated replication, thereby preventing the generation of mitochondria lacking DNA. Comprehensive investigations about the impact of mitochondrial damage on the functionality of cryopreserved sperm have been conducted in domestic animals. These studies point to oxidative stress as a key factor underlying the diminished survival and competence of cryopreserved sperm, posing a significant concern due to its correlation with reduced fertilization rates and in vitro embryo development [51]. In a recent study, supplementation of the freezing solution for human sperm with an antioxidant (myoinositol) attenuated the loss of both motility and vitality, as well as of mitochondrial activity in sperm from normozoospermic men [52].

Finally, the present study revealed not only an impact of cryopreservation on the percentage of intact acrosome but also a notable reduction in phospho-tyrosine proteins in sperm from males aged over 42 years as compared to younger individuals, with a further decline observed in frozen/thawed sperm from older men. The elevation in tyrosine phosphorylation represents a significant event commonly observed during capacitation, and this process can be influenced by the cryopreservation procedure. Indeed, several studies, most of them conducted in animals, suggest that cryopreservation induces the onset of premature capacitation [52,53]. Therefore, the significant modification in the levels of both reacted acrosome, and P-Tyr proteins observed in frozen/thawed sperm of older men can be considered tangible indicators of cryogenic damage, towards which advanced paternal age appears to be a significant contributing factor [50]. We further noted a significant decrease in phospho-Tyr staining in the tail of spermatozoa from older males; both in fresh and F/T conditions. The tyrosine phosphorylation staining in both the neck and the tail has been documented as a pattern indicative of sperm activation [33]. However, it is important to note that a direct association between the phospho-Tyr pattern and fertilization outcomes cannot be conclusively established.

## 5. Conclusions

Comprehending the interactions among paternal age, cryopreservation, and the molecular processes regulating sperm developmental competence is crucial in deciphering the complexities of male reproductive health and improving the outcomes of assisted reproductive techniques. Herein, we provide novel evidence that APA increases the sperm cell’s vulnerability to cryopreservation-derived threats to different aspects of sperm integrity/functionality. Additional research is needed to elucidate the precise molecular mechanisms governing these phenomena and their impact on fertility and reproductive outcomes.

## Figures and Tables

**Table 1:** List of patients (n = 200) characteristics and seminal values of the two study groups. Data are expressed as mean ± SD. * *p* < 0.05 ** *p* < 0.001 *** *p* < 0.0001.

**Parameters**	**Non-APA**	**APA**	***p* Value**
nº of samples	105	95	-
Age (years)	27.75 ± 6.425	45.35 ± 3.093	**<0** .0001 ***
BMI	22.5 ± 2.2	23.2 ± 1.8	**>0.05**
**Fresh semen**			
Seminal volume (mL)	2.997 ± 1.631	2.25 ± 0.8775	0.0219 *
Vitality (%)	81.18 ± 7.960	78.33 ± 10.60	0.2729
Number of spermatozoa (×10^6^)/mL	72.83 ± 45.95	65.03 ± 33.69	0.4119
Total number of spermatozoa (×10^6^)	200.5 ± 128.3	130.1 ± 70.10	0.0067 **
Progressive motility (%)	56.78 ± 12.23	50.14 ± 12.22	0.0231 *
Total motility (%)	61.50 ± 8.856	57.35 ± 10.14	0.0666
Morphologically normal sperm (%)	9.278 ± 4.193	6.703 ± 2.971	0.0036 **
**Frozen semen**			
Motility (%)	8.556 ± 8.801	5.638 ± 6.298	0.1249

## Data Availability

The authors will provide raw data upon request.

## Supplementary Materials

The following supporting information can be downloaded at https://www.mdpi.com/article/10.3390/cells13070625/s1.
