# Supplementary material for: Paternal Age Amplifies Cryopreservation-Induced Stress in Human Spermatozoa
Source: Cells. 2024 Apr 4;13(7):625. doi: 10.3390/cells13070625 (PMC11011712; doi:10.3390/cells13070625)
Supplement: Supplementary file 1 [file cells-13-00625-s001.zip › cells-2906762-supplementary.pdf]

Supplementary Table S1. List of antibodies used in this study.

Antigen	Donor Species	Dilution		Manufacturer	RRID
		IF	WB		
Primary antibodies					
P-Tyr	Mouse	1:50	1:250	Santa Cruz Biotechnology	AB_628123
Tubulin	Mouse	-	1:1000	Sigma Aldrich	AB_628123
Secondary antibodies					
Anti-Mouse IgM FITC	Goat	1:400	-	Sigma Life Science	AB_259799
Anti-Mouse-IgG HRP	Goat	-	1:2000	Bio-Rad Laboratories	AB_609692
